# Identifying the Profile of Orthorexic Behavior and “Normal” Eating Behavior with Cluster Analysis: A Cross-Sectional Study among Polish Adults

**DOI:** 10.3390/nu12113490

**Published:** 2020-11-13

**Authors:** Anna Brytek-Matera, Anna Staniszewska, Souheil Hallit

**Affiliations:** 1 Institute of Psychology, University of Wroclaw, Dawida 1, 50-527 Wroclaw, Poland; 2 Department of Experimental and Clinical Pharmacology, Medical University of Warsaw, 02-091 Warsaw, Poland; astaniszewska@wum.edu.pl; 3 INSPECT-LB: Institut National de Santé Publique, Épidémiologie Clinique et Toxicologie-Liban, Beirut, Lebanon; souheilhallit@hotmail.com; 4 Faculty of Medicine and Medical Sciences, Holy Spirit University of Kaslik (USEK), Jounieh, Lebanon

**Keywords:** orthorexia nervosa, eating behaviors, eating disorder pathology, physical activity, obsessive-compulsive disorder symptoms

## Abstract

Although the amount of research about orthorexia nervosa (ON) has grown in the last two decades, to date, research on ON remains inconsistent. More is known about some behavioral characteristics of ON and its prevalence but nothing is known about the profile analysis behind this pathological eating behavior maintenance. Therefore, the objective of the present study was to determine the profiles of the participants in terms of eating behaviors, eating disorder psychopathology, obsessive-compulsive disorder symptoms and physical activity as well as check their association with ON. The sample was composed of 229 Polish female and male adults. Our findings showed three clusters and four-related factors (obsessive-compulsive disorder features; inappropriate eating and body-related behaviors; psychological and affective traits of eating disorders; perfectionism and behaviors associated with weight maintenance or weight loss). In our sample, a higher percentage of adults belonging to cluster 1 had no ON, whereas a higher percentage of adults belonging to cluster 3 had ON. Our results emphasize the possibility to target pathological eating behaviors and obsessive-compulsive disorder (OCD) symptoms in ON in psychological intervention.

## 1. Introduction

Healthism places an emphasis on personal responsibility for health which is to be attained primarily through the modification of lifestyles [1]. Wherefore, healthism shapes numerous behaviors and practices surrounding health, body, exercise, and dieting that can redound to both improving one’s habits and promoting unhealthy habits [2]. Healthy eating behaviors are characterized by increased selection/consumption of fruits and/or vegetables, increased selection of nutrient-dense foods, or decreased selection of low-nutrient, and energy-dense foods [3]. It is difficult to recognize when healthy eating behaviors and healthy lifestyles become obsessional and problematic.

One of the disordered eating patterns is orthorexia nervosa (ON). It is characterized by obsessive thoughts and compulsive behaviors concerning healthy eating that includes rigidly following a restrictive “healthy” diet (that the individual believes to be healthy and pure, with strict avoidance of foods believed to be unhealthy) for achieving optimal health (and/or to avoid illness) [4,5]. There is still a great deal of controversy over whether ON should be considered a distinct disorder [6], a variant of other disorders (eating disorder and/or obsessive-compulsive disorder) [7] or a new lifestyle phenomenon rather than a disorder [8].

Although ON is not classified as a formal diagnostic category (neither in DSM-5 nor in ICD-11), there is initial evidence that ON shares overlapping features with eating disorders, especially anorexia nervosa (AN): eating preoccupation, dietary restriction, intense anxiety regarding certain food and their avoidance, need for control, obsessive-compulsive personality traits (e.g., perfectionism, rigidity) [6,9]. The most significant difference between ON and AN is the motivation for disordered eating: being health vs. weight loss as well as the obsession about food: quality vs. quantity [10]. Excessive preoccupation with losing weight, extreme fear of gaining weight and body size overestimation, the main features of AN, have not been diagnosed in individuals with ON [9].

According to available data, ON and obsessive-compulsive disorder (OCD) exhibit similar thinking patterns (obtrusive obsessions, e.g., thoughts on preparing the food) and behavioral patterns (repeating behaviors, e.g., ritual preparation of food) that disturb the social functioning of the individual through the need to adhere to their strictly defined dietary patterns, which significantly impairs life quality [11]. Despite these existing similarities, studies also have pointed out the discrepancy between ON and OCD: occurrence of egosyntonic (ON) vs. egodystonic thoughts (OCD) [6]. Previous studies have found a behavioral and symptomatology overlap between ON and OCD, e.g., [6,11,12,13,14,15,16,17,18,19,20]. Moreover, neurocognitive deficits (e.g., impairments in external attention) [16] and cognitive distortion (e.g., magical beliefs about food) are similar in ON and OCD (and AN) which may indicate analogous brain dysfunction in these individuals [21].

Recent research reveals that increased ON symptomatology corresponds to a high level of physical activity [12]. Several studies have found a significant positive relationship between ON symptomatology and the frequency of physical activity [13,14,16]. The recent research has shown that greater ON symptomatology was associated with more time spent on both aerobic and strength-training exercises as well as with greater levels of exercise addiction and compulsion [14], which is consistent with the results of one other study finding a significant relationship between ON and exercise addiction in a sample of fitness center members [15].

The majority of prior research has focused on epidemiological studies in non-clinical samples [22,23,24,25,26] using different questionnaires (e.g., the ORTO-15 test [27]; Eating Habits Questionnaire, EHQ [28]; the Düsseldorf Orthorexia Scale, DOS [29]; the Orthorexia Nervosa Inventory; ONI [30] and differential diagnosis). The published range of ON prevalence estimates varies considerably in the general population. Using the ORTO-15 test, ON prevalence rates range from 6% to nearly 90% [30]. While using the DOS, ON incidence ranges from 1% to 8% [31]. Such diversity of ON prevalence could result from the difference in the psychometric quality of the questionnaires [32,33] and lack of an optimal cut-off value (e.g., a cutoff score of 35 based on the ORTO-15 [27]; a cutoff score of 30 or higher based on the DOS [29]; a cuttoff score of 72 based on the ONI [31]). Although the ORTO-15 has good predictive validity [27], the lack of basic psychometric properties and the internal consistency has been criticized [32]. Psychometric flaws of the ORTO-15 indicate that this method should be replaced by another measurement. Recent research [32] has shown that to obtaining stronger evidence, researchers should use the DOS or the EHQ (internally reliable self-report instruments).

Although the amount of research about ON has grown in the last two decades, to date, the literature on ON is dominated by descriptive and anecdotal data, frequently with inconsistent results [4]. More is known about some behavioral characteristics of ON but nothing is known about the profile analysis behind this pathological eating behavior maintenance. Based on the previous studies (e.g., [6,9,11,12,13,14,16,29,30]) showing the link between ON and eating behaviors, eating disorder psychopathology, obsessive-compulsive disorder symptoms and physical activity, we decided to map those patterns and investigate their association with ON because relatively little is known about specific behaviors in ON and how they relate to different (psychological and behavioral) features. It is currently unclear whether these variables are associated with different types of varying (“normal” or disturbed) eating patterns (related or not related with ON). In addition, our study represents a first attempt to explore the typology of female and male adults based on their eating behavior which is or is not linked to ON. We assume that ON behavior (or its absence) can vary depending on investigated patterns. Given the complex nature of ON, it is worth identifying individuals with varying (“normal” or disturbed) eating patterns profiles in terms of their eating behaviors, eating disorder psychopathology, obsessive-compulsive disorder symptoms and physical activity. Since there is still a lack of a univocal definition as well as criteria in the existing literature, an ON core profile is needed. This profile will permit us to investigate patterns that are important in understanding (“normal” or disturbed) eating patterns related or not related to ON.

Person-centered approaches (also called the idiosyncratic approach) are used to detect the dynamics of emergent sub-populations in a sample (classification of similar individuals into unique sub-populations) based on a set of chosen variables and it is appropriate for examining research questions and hypotheses aimed at categorizing subjects into common subpopulations based on substantive variables and understanding the relations of these subpopulations with predictors, correlates, or outcomes [34]. Cluster-analysis has traditionally been the most commonly used method of choice in person-centered research. Person-specific analyses result in a unique model or set of parameters for each subject, which can then be grouped based on similarities among the individuals’ model [34]. Thus, the person-centered approach results in multiple sets of parameters that more narrowly detail the identified subpopulations (but the results may be more difficult to interpret as each subpopulation results in these differing parameters) [34].

The objective of the present study was to determine the profiles of the Polish adults in terms of eating behaviors, eating disorder psychopathology, obsessive-compulsive disorder symptoms and physical activity and check their association with ON. We focused on mapping the pattern of eating behaviors, eating disorders, physical activity and obsessive-compulsive disorder in ON using a cluster analysis (to investigate which mentioned variables could contribute to a lack of ON versus a risk of ON vs. having ON). We aimed at investigating (1) what emergent groups (clusters) can be identified through the ON and (2) which variable values have a significant influence on participants concerning their ON categories. The expected result of this study was forming the three clusters within ON and associated variables (we hypothesized that adults can be grouped into three distinct subgroups based on their ON behavior, eating behaviors, eating disorders, physical activity and OCD symptoms).

## 2. Materials and Methods

### 2.1. Participants and Study Design

A total of 230 participants (175 females, 55 males; Mage = 26.52 ± 7.65; age range: 18–60) were recruited through convenience sampling through several universities, companies and health centers in Poland. Of the 313 individuals who received the questionnaires, 254 (81.15%) returned them. Finally, from those, 230 questionnaires (73.48%) were recognized as completed to provide reliable data.

The survey was administered via the Internet. Participants received notice about the research with the announcement including the online link to the study. Interested individuals were invited to visit a website that directed them to the consent form, information form (purpose of the current study, anonymity, voluntariness of consent to research), and questionnaires. All participants offered their informed consent before starting the survey and responded voluntarily to the survey. There was no financial compensation for participating in the study.

The present study has been approved by the SWPS University of Social Sciences and Humanities Human Research Ethics Committee (No. WKEB59/05/2019). All procedures performed in our study were in accordance with the 1964 Helsinki declaration (adopted by the 18th World Medical Association General Assembly, Helsinki, Finland) and its later amendments or comparable ethical standards.

### 2.2. Minimal Sample Size Calculation

The minimal sample size was calculated based on a previous study [35] that showed a correlation coefficient between orthorexia (assessed by the DOS scale) and bulimia (assessed by the EDI scale). Based on a correlation coefficient of 0.195, the minimal sample size calculated was 211 [36].

### 2.3. Measures

#### 2.3.1. The Düsseldorf Orthorexia Scale (DOS)

The Düsseldorf Orthorexia Scale is a 10-item unidimensional questionnaire assessing and screening ON. A score of 30 or higher indicates the presence of ON. A score between 25 and 29 demonstrates a risk of developing ON, while a total score of less than 25 identifies the absence of ON [29]. The DOS shows good internal consistency (Cronbach’s alpha = 0.84) and good test-retest reliability (r = 0.67 to 0.79, *p* = 0.001) [19]. In the current sample, Cronbach’s alpha coefficient of the DOS was 0.84. In the present study, we used the Polish version of this questionnaire (PL-DOS) [37].

#### 2.3.2. The Three-Factor Eating Questionnaire (TFEQ-R18)

The TFEQ-R18 [38] is an 18-item self-report questionnaire assessing three different aspects of eating behaviors: (1) cognitive restraint—a conscious restriction of food intake in order to control body weight or to promote weight loss, (2) uncontrolled eating—a tendency to eat more than usual due to a loss of control over intake accompanied by subjective feelings of hunger, and (3) emotional eating—an inability to resist emotional cues, the tendency to eat in response to negative emotions. In the present study, we used the Polish version of the TFEQ-R18 [30], which has demonstrated satisfactory levels of internal reliability (α = 0.78 for cognitive restraint, α = 0.84 for uncontrolled eating and α = 0.86 for emotional eating). In the present sample, Cronbach’s alpha coefficients of the TFEQ-R18 ranged from 0.78 to 0.88.

#### 2.3.3. The Eating Disorder Inventory (EDI)

The EDI [39] is a 64-item widely used self-report questionnaire assessing the presence of eating disorder psychopathology and related features: (1) drive for thinness—an excessive concern with dieting, preoccupation with weight and entrenchment in an extreme pursuit of thinness, (2) bulimia—a tendency toward episodes of uncontrollable overeating (bingeing) and followed by the impulse to engage in self-induced vomiting, (3) body dissatisfaction—the belief that specific parts of the body associated with shape change or increased “fatness” at puberty are too large, (4) ineffectiveness—the feelings of general inadequacy, insecurity, worthlessness and the feeling of not being in control of one’s life, (5) perfectionism—an excessive personal expectations for superior achievement, (6) interpersonal distrust—a sense of alienation and a general reluctance to form close relationships, (7) interoceptive awareness—a lack of confidence in recognizing and accurately identifying emotions and sensations of hunger or satiety, and (8) maturity fears—a wish to retreat to the security of the preadolescent years because of the overwhelming demands of adulthood. In the present study, we used the Polish version of the EDI [40], which has shown a satisfactory internal consistency (α = 0.65 to 0.92). In the current sample, Cronbach’s alpha coefficients of the EDI subscales ranged from 0.50. to 0.91.

#### 2.3.4. The International Physical Activity Questionnaire (IPAQ)

The short form of the IPAQ (7-items) [41] assesses three specific types of activity: walking, moderate-intensity activities and vigorous-intensity activities in the last seven days. Subjects are asked to answer in order to record the number of days (frequency) and the number of minutes per day (duration) of their participation in all kinds of physical activity during the last seven days as well as the time spent sitting during an average weekday. The IPAQ has been validated and used widely [42,43] given its practicability in large samples. The adaptation of IPAQ-short into Polish followed the IPAQ committee guidelines (https://sites.google.com/site/theipaq/questionnaire_links; [44]). We used the combined total physical activity score: total physical activity MET (metabolic equivalent)-minutes/week = sum of walking + moderate + vigorous MET-minutes/week scores.

#### 2.3.5. The Obsessive-Compulsive Inventory—Revised (OCI-R)

The OCI-R [45] is an 18-item self-report questionnaire assessing obsessive-compulsive disorder symptoms: (1) washing, (2) ordering, (3) hoarding, (4) mental neutralizing, (5) obsessing and (6) checking. The recommended cutoff score is 21, with scores at or above this level indicating the likely presence of OCD. The original version displayed evidence of good reliability and validity indices of the OCI-R, showing strong convergence with established measures of OCD, moderate to high internal consistency across the six subscales, and adequate to high test-retest stability [45]. In the present study, we used the Polish version of the OCI-R [46], which has presented adequate test reliability for the full scale and subscales scores, high internal consistency (0.62 < Cronbach’s α < 0.85) and confirmed satisfactory convergent and divergent validity [47]. In the present sample, Cronbach’s alpha coefficients of the OCI-R ranged from 0.64 to 0.88.

In addition, the participants were asked questions regarding socio-demographic characteristics (age, weight and height) and dietary patterns (omnivorous, vegetarian).

## 3. Results

### 3.1. Statistical Analysis

Statistical analyses were carried out using the Statistical Package for Social Sciences 25.0 (SPSS, Chicago, IL, USA). Patterns among specific samples can be concluded from the factor and cluster analyses. An exploratory factor analysis was conducted as the first step to classify patterns of the multiple factors associated with ON; the extraction was done using a promax rotation since the factors were correlated. Sample adequacy was guaranteed using the Kaiser–Meyer–Olkin (KMO) index and Bartlett’s Chi-square test of sphericity. Factors with an Eigenvalue >1 were retained. Items with factor loading >0.4 were considered as belonging to a factor. A cluster analysis, using the K-mean method, was conducted next using the results obtained in the exploratory factor analysis, in order to classify participants according to their patterns (into three clusters). The Chi-square test was used to compare DOS categories with categorical variables, whereas the ANOVA test was used to compare three or more means. Three multinomial regressions were conducted taking the ON categories as the dependent variable; in the first one, each scale was entered as a separate independent variable; in the second one, the factors obtained from the factor analysis were entered as independent variables, whereas in the third one, the clusters obtained from the cluster analysis were entered as independent variables.

The DOS total score did not follow a normal distribution as verified by the Shapiro–Wilk test; therefore, non-parametric tests were used. The Mann–Whitney and Kruskal–Wallis tests were used to check for an association between two and three or more means respectively, whereas the Spearman correlation test was used to check for an association between continuous variables. A forward linear regression analysis, taking the DOS total score as the dependent variable, was conducted; all variables that showed a *p* < 0.2 were considered as independent variables in order to eliminate confounding variables as much as possible. All models were adjusted over sociodemographic variables taken as covariates. *p* < 0.05 was considered significant.

### 3.2. Characteristics of the Study Population

The sample was composed of 175 Polish women (76.1%) and 55 men (23.9%) with ages ranging between 18 and 60 years (mean 26.52 ± 7.65 years). The entire group of participants had a mean body mass index (BMI) value (24.84 kg/m^2^ ± 5.31) in the range of normal weight (from 18.5 to 24.9 kg/m^2^). One hundred thirty-eight participants (60.0%) indicated an omnivore diet and 92 (40.0%) reported to adhere to a vegetarian diet.

In the whole sample of adults, 7 (3.0%) participants exhibited traits of ON, 13 (5.7%) participants were at risk of ON and 210 (91.3%) participants presented “normal” eating behavior (no risk of developing ON). Descriptive statistics are presented in Table 1.

### 3.3. Exploratory Factor Analysis for the Methods Used in the Present Study

The factor analysis for the scales’ scores was run over the whole sample. All items could be extracted and converged over a four-factor solution that had an Eigenvalue over 1, explaining a total of 63.40% of the variance (Factor 1: obsessive-compulsive disorder features; Factor 2: inappropriate eating and body-related behaviors; Factor 3: psychological and affective traits of eating disorders; Factor 4: perfectionism and behaviors associated with weight maintenance or weight loss); KMO = 0.854; Bartlett’s test of sphericity *p* < 0.001. According to the promax rotated matrix, the components are summarized in Table 2.

### 3.4. Cluster Analysis

**Figure 1:**
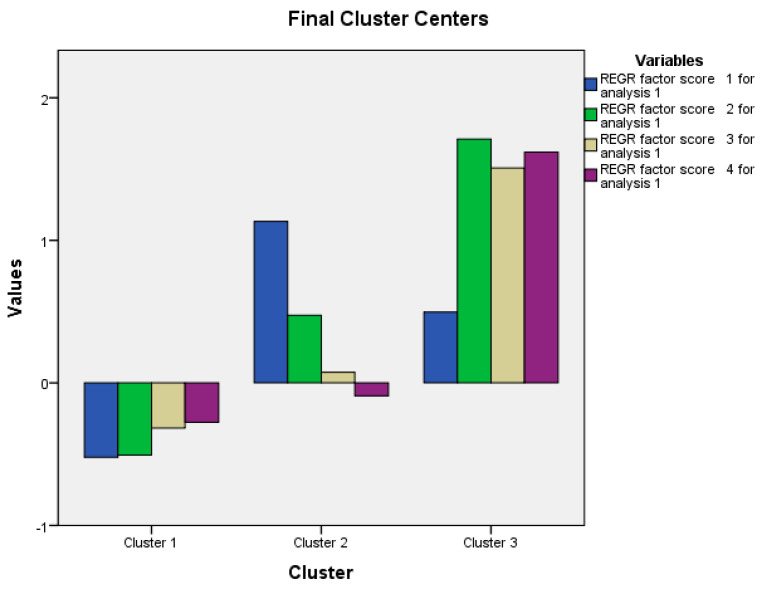
Illustration of four factors in the three profiles defined in the cluster analysis. REGR: Regression.

A cluster analysis was used to identify profiles of individuals within our sample. We found three clusters (Table 3; Figure 1). Cluster 1 includes adults with low obsessive-compulsive disorder (OCD) features, low inappropriate eating behaviors, low psychological and affective traits of eating disorders (ED) and low perfectionism and behaviors associated with weight maintenance or weight loss. Cluster 2 includes individuals with high OCD features, moderate inappropriate eating behaviors, moderate psychological and affective ED traits and moderate perfectionism and behaviors associated with weight maintenance or weight loss. Cluster 3 includes subjects with moderate OCD features, high inappropriate eating behaviors, high psychological and affective ED traits and high perfectionism and behaviors associated with weight maintenance or weight loss.

### 3.5. Bivariate Analysis

In order to investigate factors associated with the three ON categories among adults (absence of ON, at risk of ON, presence of ON) a bivariate analysis was used (Table 4). The results of the bivariate analysis of factors associated with the DOS categories showed that a higher percentage of people belonging to cluster 1 had no ON, whereas a higher percentage of people belonging to cluster 3 had ON. Furthermore, higher cognitive restraint, drive for thinness, hoarding and total OCI-R scores were significantly found in participants at risk of ON. Finally, a higher ineffectiveness was found in participants with ON.

The results of the bivariate analysis of factors associated with the DOS total score showed that a higher mean DOS score was found in participants belonging to cluster 3 compared to the other two clusters. Furthermore, higher DOS scores were significantly associated with higher cognitive restraint, uncontrolled eating, emotional eating, drive for thinness, bulimia, interoceptive awareness, ordering, mental neutralizing, washing, obsessing, checking and total OCI-R score (Table 5).

### 3.6. Mulivariable Analysis

Multinomial logistic regression was used to assess factors (independent variables) associated with a nominal/categorical dependent variable (in our case DOS categories). The results of the multinomial logistic regression, acknowledged as being at risk of ON vs. absence of ON as the dependent variable and each score as an independent variable, showed that higher cognitive restraint (aOR = 1.12), higher drive for thinness (aOR = 1.17) and more hoarding (aOR = 1.30) were significantly associated with higher odds of being at risk of ON, whereas higher ineffectiveness (aOR = 0.74) was significantly associated with lower odds of being at risk of ON (Table 6, Model 1).

None of those variables were significantly associated with the presence of ON compared to its absence (Table 6, Model 2).

The results of the multinomial logistic regression, acknowledged as being at risk of ON vs. absence of ON as the dependent variable and the four factors obtained in the factor analysis as independent variables, showed that higher Factor 1 scores (more obsessive-compulsive disorder features) (aOR = 1.88), and higher Factor 4 scores (more perfectionism and behaviors associated with weight maintenance or weight loss) (aOR = 2.05) were significantly associated with higher odds of being at risk of ON (Table 7, Model 1).

None of those factors was significantly associated with the presence of ON compared to its absence (Table 7, Model 2).

The results of the multinomial logistic regression, acknowledged as being at risk of ON vs. absence of ON as the dependent variable and the clusters as independent variables, showed that belonging to cluster 2 vs. cluster 1 (aOR = 4.31) was significantly associated with higher odds of being at risk of ON (Table 8, Model 1).

None of those clusters were significantly associated with the presence of ON compared to its absence (Table 8, Model 2).

The results of a forward linear regression, taking the DOS total score as the dependent variable, showed that a higher drive for thinness (β = 0.22), higher cognitive restraint (β = 0.45) and higher mental neutralizing (β = 0.39) were significantly associated with higher orthorexia nervosa (higher DOS scores) (Table 9).

## 4. Discussion

Person-specific analysis, conducted in the present study, resulted with three clusters showing groups of adults according to their ON related-behaviors. We hypothesized that within our Polish adult sample, the cluster analysis would reveal three groups of adults with distinct patterns of comorbid behaviors. In our analysis, the largest cluster (cluster 1; 63.75% of the sample) was characterized by low OCD features (ordering, mental neutralizing, washing, checking, obsessing and hoarding), low inappropriate eating and body-related behaviors (emotional eating, uncontrolled eating, bulimia, drive for thinness and interoceptive awareness), low psychological and affective ED traits (ineffectiveness, interpersonal distrust, maturity and fear and body dissatisfaction) and low perfectionism and behaviors associated with weight maintenance or weight loss (cognitive restraint, perfectionism and physical activity). Individuals in cluster 2 (24.02% of the sample) were characterized by high OCD features, moderate inappropriate eating and body-related behaviors, moderate psychological and affective ED traits and moderate perfectionism and behaviors associated with weight maintenance or weight loss. Last, the smallest cluster (cluster 3; 12.23% of the sample) was characterized by moderate OCD features, high inappropriate eating and body-related behaviors, high psychological and affective ED traits and high perfectionism and behaviors associated with weight maintenance or weight loss.

Our results demonstrated that adults who belonged to cluster 1 showed “normal” eating behavior and had no risk of ON, whereas adults who belonged to cluster 3 had ON. Belonging to cluster 2 (vs. cluster 1) was significantly associated with higher odds of being at risk of ON among adults who participated in the present study. This could indicate that higher OCD features, moderate inappropriate eating and body-related behaviors, moderate EDs symptoms and moderate low perfectionism and behaviors associated with weight maintenance or weight loss are associated with being at risk of developing ON. While, moderate OCD and high inappropriate eating and body-related behaviors, high ED symptoms and high perfectionism and behaviors associated with weight maintenance or weight loss are related to having ON. These findings are consistent with the results of the studies pointing out that ON is more linked to ED than to OCD [7]. The recent study [7] has found that ON symptoms were related to perfectionism, higher body weight and shape concerns and with prioritizing weight above health with respect to food selection. Another study [48] has demonstrated ON to considerably overlap with pathological eating, with 78% of ON cases (as compared to 29% in non-ON) showing above-threshold symptoms of an ED. In addition, this study has also shown that 30% of ON individuals (as compared to 11% in non-ON) showed considerable OCD symptoms.

In our sample, a higher level of OCD features (factor 1) and higher perfectionism and behaviors associated with weight maintenance or weight loss (factor 4) were associated with higher odds of being at risk of ON. Previous studies have shown a positive association between ON and OCD [18,19,49], perfectionism [20,50], cognitive restraint [51] and physical activity [14].

Our study showed that a higher conscious restriction of food intake in order to control body weight or to promote weight loss, higher excessive concern with dieting as well as a preoccupation with weight and entrenchment in an extreme pursuit of thinness and higher total OCI-R scores were found in adults at risk of ON. Whereas higher feelings of general inadequacy, insecurity, worthlessness and the feeling of not being in control of participants’ life were significantly associated with lower odds of being at risk of ON. The recent review on psychosocial ON factors [52] has found that perfectionism, OC traits, psychopathology, disordered eating, history of an ED, dieting, negative body image and drive for thinness were positively related to greater ON. The recent studies conducted among non-clinical samples have demonstrated that drive for thinness [7,22] and body dissatisfaction [53] were more likely among individuals with ON than those without ON or low level of ON. According to Barthels et al. [53] ON and ED share psychopathological features (body dissatisfaction) and this suggests a close relationship between both disorders.

Our findings showed that more hoarding was found in adults at risk of ON. Deficits in cognitive processes, maladaptive beliefs and maladaptive behavioral patterns are considered to underlie pathological hoarding [54]. Individuals who hoard have abnormalities in the specific brain regions associated with executive functioning, impulse control, and processing of reward value [54]. At present, there is lack of research assessing if individuals with ON exhibit analogous patterns. Only one existing neuropsychological study [20] evaluated the cognitive profile of ON (targeting the cognitive domains to be affected in AN and OCD), including executive functioning, attention, verbal long-term memory and visuospatial functioning). ON severity was negatively associated with executive functioning as assessed via self-report. In addition, individuals with ON reported more broadband executive dysfunction using a neuropsychological battery. Moreover, individuals with ON received worse results in domains of set-shifting, external attention, and working memory compared to participants without ON. Koven and Senbonmatsu [20] claimed that ON symptoms are independently associated with essential aspects of executive functioning (set-shifting, self- monitoring, and working memory) for which OCD and AN profiles already neuropsychological overlap.

Studies have demonstrated that hoarding develops as a result of conditional emotional responses to various thoughts and beliefs [55]. It has been suggested that several types of deficits (information processing, beliefs about emotional attachment to possessions, emotional distress, and avoidance behaviors) are contributors to hoarding [55]. A recent study [56] has demonstrated that difficulties identifying and regulating emotions were associated with symptoms of ON. Individuals with high ON tendencies had more difficulties identifying and accepting their feelings, and resisting impulses, engaging in goal-directed behaviors and finding the right strategies when upset compared to people with low ON tendencies. It has been hypothesized that ON behavior can be considered as a coping strategy in order to feel ‘perfect’ and in control [57] in these participants who have poor emotion regulation abilities [56]. It is possible that people with ON tendencies tend to perceive themselves to be more ‘out of control’ when experiencing negative emotions. Regardless of whether losing control is objectively observed or subjectively perceived, the use of very thorough and obsessive food habits could thus serve to increase levels of perceived control in order to cope with difficult feelings [56]. It is worth adding that a higher ineffectiveness was found in our sample with ON.

A number of limitations should be borne in mind. With a small sample size in cluster 3 (having ON) the findings cannot be extrapolated to all individuals with ON. Moreover, the number of participants who meet the criteria for ON or for being at risk of ON is small and represents less than 10% of the total sample. These results are in line with other studies showing that ON prevalence (used the DOS) among a general population sample ranges from 2.3% [58] to 8.4% [59]. Future research using claster analysis should be conducted on a large cohort from different samples (both clinical and non-clinical). Larger sample sizes (>500) are often deemed most appropriate for mixture models [34].

As with any research, the characteristics of our clusters are limited to our data and setting. There may be other individual characteristics which we did not include (e.g., depression, social functioning disturbances, self-control). The next limitation of our study is associated with a disproportionately high ratio of female adults. The cross-sectional study design is unable to identify directions of causality. A selection bias also is present because of the convenience sampling technique followed.

Our results suggest that being at risk of ON is more classified on the OCD spectrum, whereas ON is more classified on the ED spectrum. Since the motivation for specific food choices and eating behaviors differ in ON and ED distinguishing both of these disorders is necessary from a therapeutic perspective [48]. The profile of individuals being at risk of ON and having ON should be explored in detail in order to develop a relevant psychological intervention. Moreover, the identification of modifiable risk factors for abnormal eating patterns (at risk of ON and having ON) can help create targeted prevention strategies for ON in the adult population.

## 5. Conclusions

In the present study, we used a cluster analysis to identify distinct subgroups of Polish adults with and without ON and specific co-occurring behaviors. To the best of our knowledge, the present study was the first to investigate the profile analysis of different types of varying (“normal” or disturbed) eating patterns (related or not related with ON).

A cluster analysis can be used to identify separate groups of individuals with specific combinations of comorbid behaviors. This method can be used to develop targeted psychological interventions designed to improve health and eating outcomes. Future research could focus on collecting data from larger sample (both clinical and non-clinical) for the purpose of assessing the profile analysis of ON. Identifying individuals with ON based on their actual pattern of behaviors has the potential to be used in the psychological intervention focusing on improving their health and eating behaviors.

## Figures and Tables

**Table 1:** Mean scores (M) and standard deviations (SD) for the sample.

Variables	M ± SD
Age	26.52 ± 7.65
Body Mass Index	24.84 ± 5.31
Orthorexia nervosa (DOS)	17.66 ± 5.19
Cognitive restraint (TFEQ-R18)	10.34 ± 2.82
Uncontrolled eating (TFEQ-R18)	9.19 ± 5.80
Emotional eating (TFEQ-R18)	3.44 ± 2.64
Drive for thinness (EDI)	4.29 ± 4.81
Bulimia (EDI)	2.65 ± 4.39
Body dissatisfaction (EDI)	8.27 ± 7.36
Ineffectiveness (EDI)	5.71 ± 5.12
Perfectionism (EDI)	5.06 ± 4.09
Interpersonal distrust (EDI)	4.20 ± 4.24
Interoceptive awareness (EDI)	4.49 ± 5.29
Maturity fear (EDI)	6.33 ± 5.47
Total physical activity (IPAQ)	2342.91 ± 2877.45
Hoarding (OCI-R)	3.19 ± 2.76
Ordering (OCI-R)	3.47 ± 2.88
Mental neutralizing (OCI-R)	1.52 ± 2.40
Washing (OCI-R)	2.28 ± 2.42
Obsessing (OCI-R)	2.11 ± 2.69
Checking (OCI-R)	3.80 ± 3.11
Total OCI-R	16.36 ± 12.62

DOS: Düsseldorf Orthorexia Scale; TFEQ-R18: Three-Factor Eating Questionnaire; EDI: Eating Disorder Inventory; IPAQ: International Physical Activity Questionnaire; OCI-R: Obsessive Compulsive Inventory—Revised.

**Table 2:** Exploratory factor analysis of the scales scores using the promax rotation.

Variable	Factor 1 Obsessive-Compulsive Disorder Features	Factor 2 Inappropriate Eating and Body-Related Behaviors	Factor 3 Psychological and Affective Traits of Eating Disorders	Factor 4 Perfectionism and Behaviors Associated with Weight Maintenance or Weight Loss	h^2^ Communalities
Ordering (OCI-R)	0.841				0.717
Mental neutralizing (OCI-R)	0.808				0.682
Washing (OCI-R)	0.785				0.549
Checking (OCI-R)	0.775				0.578
Obsessing (OCI-R)	0.700				0.712
Hoarding (OCI-R)	0.661				0.599
Emotional eating (TFEQ-R18)		0.992			0.750
Uncontrolled eating (TFEQ-R18)		0.956			0.718
Bulimia (EDI)		0.797			0.799
Drive for thinness (EDI)		0.500			0.683
Interoceptive awareness (EDI)		0.497			0.799
Ineffectiveness (EDI)			0.801		0.773
Interpersonal distrust (EDI)			0.763		0.442
Maturity and fear (EDI)			0.745		0.523
Body dissatisfaction (EDI)			0.550		0.488
Cognitive restraint (TFEQ-R18)				0.750	0.625
Perfectionism (EDI)				0.653	0.548
Total physical activity (IPAQ)				0.644	0.427
Variance explained	33.74	14.40	8.50	6.76	

TFEQ-R18: Three-Factor Eating Questionnaire; EDI: Eating Disorder Inventory; IPAQ: International Physical Activity Questionnaire; OCI-R: Obsessive Compulsive Inventory—Revised.

**Table 3:** Results of cluster analysis.

Factor	Cluster 1 *N* = 146 (63.75%)	Cluster 2 *N* = 55 (24.02%)	Cluster 3 *N* = 28 (12.23%)
Factor 1: Obsessive-compulsive disorder features	−0.52	1.13	0.49
Factor 2: Inappropriate eating and body-related behaviors	−0.51	0.47	1.71
Factor 3: Psychological and affective traits of eating disorders	−0.32	0.07	1.51
Factor 4: Perfectionism and behaviors associated with weight maintenance or weight loss	−0.28	-0.09	1.62

**Table 4:** Bivariate analysis of factors associated with the DOS categories.

Variable	ON Categories (Based on the DOS)			*p*	Chi-Square Test
	Absence of ON (*N* = 210) Having a Score <25	At risk of ON (*N* = 13) Having a Score at 25–29	Presence of ON (*N* = 7) Having a Score ≥30		
Clusters				**0.038**	8.50
Cluster 1	138 (66.0%)	4 (20.8%)	4 (57.1%)		
Cluster 2	48 (23.0%)	6 (46.2%)	1 (14.3%)		
Cluster 3	23 (11.0%)	3 (23.1%)	2 (28.6%)		
Gender				0.443	1.90
Males	160 (76.2%)	11 (84.6%)	4 (57.1%)		
Females	50 (23.8%)	2 (15.4%)	3 (42.9%)		
Age	26.66 ± 7.57	23.54 ± 2.33	27.86 ± 14.31	0.724	-
Body Mass Index	24.82 ± 5.35	23.59 ± 4.06	27.68 ± 5.77	0.257	-
Cognitive restraint (TFEQ-R18)	10.22 ± 2.80	12.23 ± 2.97	10.57 ± 1.72	**0.042**	-
Uncontrolled eating (TFEQ-R18)	9.00 ± 5.83	9.92 ± 3.77	13.57 ± 6.65	0.109	-
Emotional eating (TFEQ-R18)	3.37 ± 2.66	4.31 ± 1.97	4.14 ± 3.13	0.358	-
Drive for thinness (EDI)	3.99 ± 4.57	7.92 ± 5.98	6.57 ± 6.75	**0.007**	-
Bulimia (EDI)	2.57 ± 4.35	1.92 ± 2.36	6.28 ± 6.97	0.393	-
Body dissatisfaction (EDI)	8.20 ± 7.22	8.46 ± 8.78	10.14 ± 9.79	0.788	-
Ineffectiveness (EDI)	5.66 ± 5.06	3.84 ± 2.19	10.57 ± 8.06	**0.017**	-
Perfectionism (EDI)	4.96 ± 4.07	6.61 ± 2.75	5.43 ± 6.40	0.134	-
Interpersonal distrust (EDI)	4.14 ± 4.29	3.77 ± 3.54	6.86 ± 3.67	0.234	-
Interoceptive awareness (EDI)	4.27 ± 5.01	5.69 ± 4.46	9.00 ± 11.16	0.188	-
Maturity fear (EDI)	6.24 ± 5.41	8.08 ± 5.39	5.71 ± 7.50	0.484	-
Total physical activity (IPAQ)	2280.91 ± 2890.06	2843.24 ± 2465.07	3264.86 ± 3355.09	0.548	-
Hoarding (OCI-R)	3.06 ± 2.74	5.07 ± 2.69	3.71 ± 2.75	**0.033**	-
Ordering (OCI-R)	3.36 ± 2.88	5.23 ± 2.52	3.57 ± 2.57	0.075	-
Mental neutralizing (OCI-R)	1.40 ± 2.32	2.92 ± 3.33	2.43 ± 2.07	0.05	-
Washing (OCI-R)	2.18 ± 2.38	3.38 ± 2.87	3.00 ± 2.24	0.161	-
Obsessing (OCI-R)	1.99 ± 2.65	3.23 ± 2.92	3.57 ± 2.99	0.093	-
Checking (OCI-R)	3.70 ± 3.10	4.69 ± 3.25	4.86 ± 3.29	0.357	-
Total OCI-R	15.70 ± 12.44	24.54 ± 13.80	21.14 ± 11.00	**0.029**	-

ON: Orthorexia Nervosa; DOS: Düsseldorf Orthorexia Scale; TFEQ-R18: Three-Factor Eating Questionnaire; EDI: Eating Disorder Inventory; IPAQ: International Physical Activity Questionnaire; OCI-R: Obsessive Compulsive Inventory—Revised. Post hoc analysis: cognitive restraint (absence of ON vs. at risk of ON *p* = 0.037); Drive for thinness (absence of ON vs. at risk of ON *p* = 0.012); Ineffectiveness (absence of ON vs. presence of ON *p* = 0.037; at risk of ON vs. presence of ON *p* = 0.015); Hoarding (absence of ON vs. at risk of ON *p* = 0.031); Total OCI-R (absence of ON vs. at risk of ON *p* = 0.042). Numbers in bold indicate significant *p*-values.

**Table 5:** Bivariate analysis of factors associated with the DOS total score.

Variable	DOS Total Score	*p*
Clusters		**0.003**
Cluster 1	16.86 ± 5.02	
Cluster 2	18.40 ± 5.03	
Cluster 3	20.36 ± 5.51	
Gender		0.111
Males	17.07 ± 6.00	
Females	17.85 ± 4.91	
Age	0.001	0.997
Body Mass Index	0.057	0.387
Cognitive restraint (TFEQ-R18)	0.395	**<0.001**
Uncontrolled eating (TFEQ-R18)	0.251	**<0.001**
Emotional eating (TFEQ-R18)	0.275	**<0.001**
Drive for thinness (EDI)	0.350	**<0.001**
Bulimia (EDI)	0.184	**0.005**
Body dissatisfaction (EDI)	0.089	0.179
Ineffectiveness (EDI)	0.066	0.317
Perfectionism (EDI)	0.070	0.289
Interpersonal distrust (EDI)	0.096	0.147
Interoceptive awareness (EDI)	0.166	**0.012**
Maturity fear (EDI)	0.049	0.464
Total physical activity (IPAQ)	-0.043	0.515
Hoarding (OCI-R)	0.129	0.050
Ordering (OCI-R)	0.218	**0.001**
Mental neutralizing (OCI-R)	0.213	**0.001**
Washing (OCI-R)	0.149	**0.023**
Obsessing (OCI-R)	0.217	**0.001**
Checking (OCI-R)	0.218	**0.001**
Total OCI-R	0.253	**<0.001**

DOS: Düsseldorf Orthorexia Scale; TFEQ-R18: Three-Factor Eating Questionnaire; EDI: Eating Disorder Inventory; IPAQ: International Physical Activity Questionnaire; OCI-R: Obsessive Compulsive Inventory—Revised. Post-hoc analysis: cluster 1 vs. cluster 3 *p* = 0.003. Numbers in bold indicate significant *p*-values.

**Table 6:** Multivariable analysis: multinomial logistic regression taking the absence of orthorexia nervosa as the reference group.

**Model 1: At Risk of ON vs. Absence of ON**			
**Variable**	***p*** ****	**aOR**	**95% CI**
Cognitive restraint (TFEQ-R18)	0.333	1.12	0.89–1.42
Drive for thinness (EDI)	**0.011**	1.17	1.04–1.32
Ineffectiveness (EDI)	**0.017**	0.74	0.58–0.95
Hoarding (OCI-R)	**0.024**	1.30	1.04–1.64
**Model 2: Presence of ON vs. Absence of ON**			
**Variable**	***p*** ****	**aOR**	**95% CI**
Cognitive restraint (TFEQ-R18)	0.995	0.99	0.74–1.34
Drive for thinness (EDI)	0.862	1.02	0.84–1.23
Ineffectiveness (EDI)	0.111	1.12	0.98–1.28
Hoarding (OCI-R)	0.808	0.97	0.72–1.29

ON: Orthorexia Nervosa; TFEQ-R18: Three-Factor Eating Questionnaire; EDI: Eating Disorder Inventory; OCI-R: Obsessive Compulsive Inventory—Revised. Numbers in bold indicate significant *p*-values. Goodness-of-fit Pearson X^2^ = 355.03; *p* = 0.998; Nagelkerke Pseudo R^2^ = 0.219. Numbers in bold indicate significant *p*-values.

**Table 7:** Multivariable analysis: multinomial logistic regression taking the absence of orthorexia nervosa as the reference group.

**Model 1: At Risk of ON vs. Absence of ON**			
**Variable**	***p***	**aOR**	**95% CI**
Factor 1: Obsessive-compulsive disorder features	**0.017**	1.88	1.12–3.16
Factor 2: Inappropriate eating and body-related behavior	0.848	0.94	0.47–1.86
Factor 3: Psychological and affective traits of eating disorder	0.259	0.66	0.32–1.36
Factor 4: Perfectionism and behaviors associated with weight maintenance or weight loss	**0.02**	2.05	1.12–3.76
**Model 2: Presence of ON vs. absence of ON**			
**Variable**	***p***	**aOR**	**95% CI**
Factor 1: Obsessive-compulsive disorder features	0.727	1.15	0.53–2.50
Factor 2: Inappropriate eating and body-related behavior	0.471	1.39	0.57–3.38
Factor 3: Psychological and affective traits of eating disorder	0.404	1.38	0.65–2.91
Factor 4: Perfectionism and behaviors associated with weight maintenance or weight loss	0.913	0.95	0.39–2.30

ON: Orthorexia nervosa. Goodness-of-fit Pearson X^2^ = 409.49; *p* = 0.892; Nagelkerke Pseudo R^2^ = 0.123. Numbers in bold indicate significant *p*-values.

**Table 8:** Multivariable analysis: multinomial logistic regression taking the absence of orthorexia nervosa as the reference group.

**Model 1: At Risk of ON vs. Absence of ON**			
**Variable**	***p***	**aOR**	**95% CI**
Belonging to cluster 2 (yes vs. no)	**0.028**	4.31	1.17–15.94
Belonging to cluster 3 (yes vs. no)	0.059	4.50	0.95–21.43
**Model 2: Presence of ON vs. Absence of ON**			
**Variable**	***p***	**aOR**	**95% CI**
Belonging to cluster 2 (yes vs. no)	0.770	0.72	0.08–6.59
Belonging to cluster 3 (yes vs. no)	0.220	3.00	0.52–17.33

Nagelkerke Pseudo R^2^ = 0.066. Numbers in bold indicate significant *p*-values.

**Table 9:** Multivariable analysis: Forward linear regression taking the DOS total score as the dependent variable.

Variable	UB	SB	*p*	95% CI
Drive for thinness	0.22	0.21	**0.002**	0.08–0.36
Cognitive restraint	0.45	0.24	**<0.001**	0.21–0.68
Mental neutralizing	0.39	0.18	**0.004**	0.13–0.65

UB: unstandardized beta; SB: standardized beta; CI: confidence interval. Nagelkerke R^2^ = 0.178. Numbers in bold indicate significant *p*-values.
